# Serum complement proteins rather than inflammatory factors is effective in predicting psychosis in individuals at clinical high risk

**DOI:** 10.1038/s41398-022-02305-1

**Published:** 2023-01-12

**Authors:** TianHong Zhang, JiaHui Zeng, JiaYi Ye, YuQing Gao, YeGang Hu, LiHua Xu, YanYan Wei, XiaoChen Tang, HaiChun Liu, Tao Chen, ChunBo Li, ChunLing Wan, JiJun Wang

**Affiliations:** 1 Shanghai Mental Health Center, Shanghai Jiaotong University School of Medicine, Shanghai Intelligent Psychological Evaluation and Intervention Engineering Technology Research Center (20DZ2253800), Shanghai Key Laboratory of Psychotic Disorders, Shanghai, 200030 PR China; 2 Department of Automation, Shanghai Jiao Tong University, Shanghai, 200240 PR China; 3 Big Data Research Lab, University of Waterloo, Waterloo, ON Canada; 4 Labor and Worklife Program, Harvard University, Cambridge, MA USA; 5 Key Laboratory for the Genetics of Developmental and Neuropsychiatric Disorders (Ministry of Education), Bio-X Center, Shanghai Jiao Tong University, Shanghai, PR China; 6 Center for Excellence in Brain Science and Intelligence Technology (CEBSIT), Chinese Academy of Science, Beijing, PR China; 7 Brain Science and Technology Research Center, Shanghai Jiao Tong University, Shanghai, PR China

**Keywords:** Predictive markers, Schizophrenia

## Abstract

Immunological/inflammatory factors are implicated in the development of psychosis. Complement is a key driver of inflammation; however, it remains unknown which factor is better at predicting the onset of psychosis. This study aimed to compare the alteration and predictive performance of inflammation and complement in individuals at clinical high risk (CHR). We enrolled 49 individuals at CHR and 26 healthy controls (HCs). Twenty-five patients at CHR had converted to psychosis (converter) by the 3-year follow-up. Inflammatory cytokines, including interleukin (IL)-1β, 6, 8, 10, tumor necrosis factor-alpha (TNF-alpha), macrophage colony-stimulating factor levels, and complement proteins (C1q, C2, C3, C3b, C4, C4b, C5, C5a, factor B, D, I, H) were measured by enzyme-linked immunosorbent assay at baseline. Except for TNF- alpha, none of the inflammatory cytokines reached a significant level in either the comparison of CHR individuals and HC or between CHR-converters and non-converters. The C5, C3, D, I, and H levels were significantly lower (C5, *p* = 0.006; C3, *p* = 0.009; D, *p* = 0.026; I, *p* = 0.016; H, *p* = 0.019) in the CHR group than in the HC group. Compared to non-converters, converters had significantly lower levels of C5 (*p* = 0.012) and C5a (*p* = 0.007). None of the inflammatory factors, but many complement factors, showed significant correlations with changes in general function and symptoms. None of the inflammatory markers, except for C5a and C5, were significant in the discrimination of conversion outcomes in CHR individuals. Our results suggest that altered complement levels in the CHR population are more associated with conversion to psychosis than inflammatory factors. Therefore, an activated complement system may precede the first-episode of psychosis and contribute to neurological pathogenesis at the CHR stage.

## Introduction

Psychosis is one of the most severe mental disorders and can cause severe functional impairments. The incidence of psychosis has been rising in China [1]. Although the etiology and pathogenesis of psychosis are not yet fully understood, it is widely accepted that dysregulation of the immunological/inflammatory system is associated with the development and subsequent progression of psychosis [2–4]. Increasing evidence of changes in cytokines throughout the course of psychosis supports the view that complement and inflammatory markers can be considered important contributors to the prediction of psychosis [5–7].

In recent years, growing evidence linking the complement system with inflammatory conditions has identified it as a contributor to various inflammatory pathologies [8]. It is evident that complement interacts with one another to induce a series of inflammatory responses and is activated via three initiating pathways (classical, alternative, and lectin) [9] in the complex conditions of acute inflammation. Previous studies have suggested that inflammatory cytokines mediate the immune system and brain, which may lead to decreased availability of serotonin and other neurotransmitters, activation of the hypothalamic-pituitary-adrenal axis, and increased oxidative stress in the brain [10, 11]. A series of studies have demonstrated that complement is a key driver of inflammation, and complement dysregulation plays a critical role in neurodevelopment, such as developmentally timed synaptic pruning in the brain, causing pathology in psychosis [12–14]. Although changes in inflammatory and complement levels might contribute to the pathogenesis of psychosis, findings concerning peripheral levels across different stages of psychosis are mixed [3, 15–19], with findings of increased and decreased levels and no difference. These differences might be associated with the inflammatory and complement biomarkers that play varied roles in the pre-morbid phases, acute phase, or during treatment in the chronic phase of psychosis.

Patients with psychosis are usually preceded by an “at risk” stage, defined as clinical high risk (CHR), in which individuals may have attenuated positive symptoms, such as hallucinations and delusions, but have a certain degree of insight [20]. Since only 20–30% of the CHR population will convert to psychosis in the following 2–3 years [21–23], in the past two decades, biomarkers such as serum cytokines and complement factors have been widely investigated with the aim of establishing objective criteria to better characterize CHR individuals who will truly convert to psychosis. Although the CHR population is thought to be highly heterogeneous [24, 25], the study of the predictive powers of the inflammatory and complement biomarkers from the CHR state to full psychosis would provide a unique opportunity to improve the efficacy of early identification and explore the role of the dysregulated immune process in psychotic episodes [26, 27].

Although differences in serum cytokines and complement factors between CHR individuals and healthy controls (HC) have been reported, previous studies have investigated inflammatory and complement biomarkers separately, so we cannot know which of the two is better at predicting the onset of psychosis. In addition, most of the samples in previous studies were patients taking medication [28, 29], and the impact of antipsychotic drugs on the immune system may be a significant confounding factor [30]. This study addresses these issues by assessing and comparing a wide range of inflammatory cytokines and complement factors in the CHR phase of psychosis and well-matched HC subjects to better understand the effects of immune dysfunction on psychosis development. More specifically, our aims were as follows:1) to compare the levels of cytokines and complement factors between CHR individuals and HC subjects; 2) to compare the levels of cytokines and complement factors between CHR converters (conversion to psychosis) and non-converters; 3) to investigate the correlation between functional and symptomatic changes and the levels of cytokines and complement factors; and 4) to examine and compare the predictive power of the levels of cytokines and complement factors in distinguishing CHR from HC and converters from non-converters.

## Methods

### Program and participants

The current study is part of the National Key R&D Program of the Ministry of Science and Technology of China (2016YFC1306800) and ShangHai At Risk for Psychosis-extended (SHARP-extended) program, consisting of 394 help-seeking first-visit participants recruited consecutively from the Shanghai Mental Health Center (SMHC) from January 1, 2016, to June 31, 2021. The SMHC is China’s largest outpatient medication management and psychotherapy-providing mental health clinic, providing care for patients in different parts of the country. The ethics committee of the SMHC approved this study. All participants provided written informed consent during the study recruitment stage. Participants younger than 18 years had their consent forms signed by their parents, and the adolescents gave verbal consent.

**Fig. 1:**
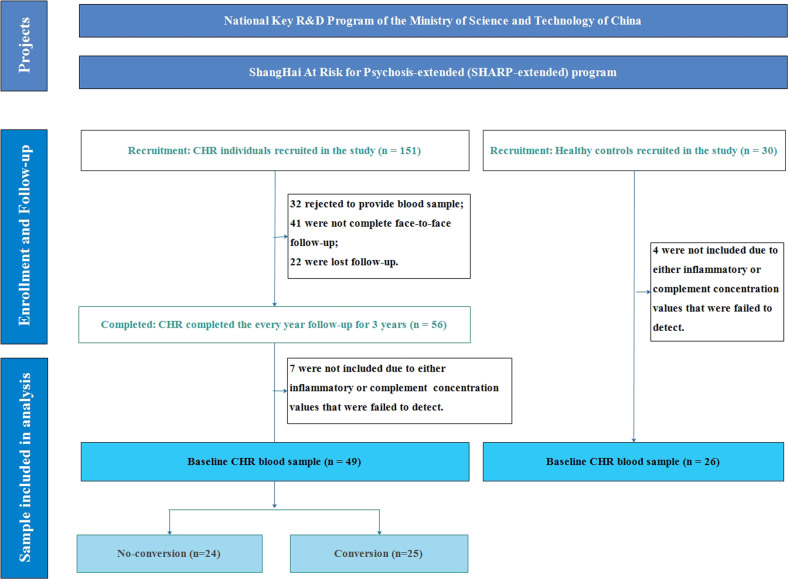
Sample flowchart. CHR Clinical high risk for psychosis, CHR-converter CHR individuals who were converted to full psychosis, HC Healthy control.

In total, 151 CHR individuals were recruited through face-to-face interviews based on the structured interview for prodromal syndromes (SIPS) [31, 32]. Thirty HCs were recruited from the community during the study period. Participants who did not provide blood samples or complete the follow-up were excluded. Finally, 49 CHR and 28 HC individuals were included (Fig. 1). Demographic and clinical characteristic comparisons were conducted between the participants included and excluded due to incomplete follow-up (Supplementary Table S1). There were more males, lower mean scores of current global assessment of function (GAF), higher mean scores of GAF drop, and higher mean scores of SIPS negative symptoms in CHR individuals included in the analysis than in those who were excluded.

Details of the study procedures, study settings, implementation of the measurements, and assessments have been reported elsewhere [21, 23, 33, 34]. A key element of our sample was that all the participants were psychotropically naïve when they entered the study and were assessed clinically. The patients had not received treatment for any psychiatric disorders. Notably, there was no history of substance abuse or dependence and smoking in this sample because substance abuse is one of the exclusion criteria for CHR recruitment.

### Clinical criteria and follow-up

SIPS [31] was used to identify individuals at CHR, including one of the following prodromal syndrome criteria: (1) brief intermittent psychotic syndrome, (2) attenuated positive symptom syndrome, or (3) genetic risk and deterioration syndrome. The SIPS consists of 19 items that assess four symptom domains: positive, negative, disorganized, and general symptoms. The GAF was used to measure the global psychological, social, and occupational functioning of the patients and to assess functional deterioration (score relative to that 12 months prior) in the SIPS interview.

Conversion to psychosis, which was determined using the criteria for the presence of psychotic symptoms [35] from the SIPS, was the major outcome of this study. Specifically, conversion was defined by the presence of a six-level positive symptom (the rating “6” refers to severe and psychotic) that was either dangerous, disorganized, or occurred at least 1 h a day on average over four days a week for at least 16 h in total. Of the 49 CHR individuals who completed the 3-year follow-up and provided serum samples, 25 were diagnosed with psychosis. The conversion outcome determination was based on face-to-face interviews.

All participants who completed the baseline assessment were followed up every year for up to 3 years. Except for those who desired no further contact, individuals were re-invited and re-assessed during face-to-face interviews every year using the SIPS, which included symptom and functioning measures. The CHR individuals and their caregivers had been told that they could contact the interviewer and study clinicians anytime to ask questions and to provide a progress report on the participants’ medical conditions during the subsequent 3 years. The conversion was determined according to the clinical information received from clinician reports and interviews with CHR individuals or their caregivers.

### Antipsychotic exposures

All the participants were psychotropically naïve when they entered the study and had not received treatment for any psychiatric disorders. As a result, the baseline levels of inflammatory cytokines and complement factors were not affected by antipsychotics. Of the 49 CHR participants who completed the follow-up and were included in the analysis, 41 (83.7%) were treated with antipsychotics and received daily olanzapine-equivalent doses (9.1 mg, with a mean administration duration of 56.2 weeks) [36]. None of the patients were treated with psychotherapy during the follow-up. The most commonly used antipsychotic in the current sample was aripiprazole (*n* = 16, 32.7%), followed by olanzapine (*n* = 13, 26.5%), amisulpride (*n* = 5, 12.2%), risperidone (*n* = 5, 12.2%), quetiapine (*n* = 1, 2.4%), and ziprasidone (*n* = 1, 2.4%). No statistically significant difference in daily olanzapine-equivalent doses was identified between the CHR-converter (mean dose = 8.5 mg/d, standard deviation (SD) = 7.5) and non-converter groups (mean dose = 7.1 mg/d, SD = 6.6). A significantly longer duration of antipsychotic administration was observed in the CHR converter (mean duration = 72.2 weeks, SD = 36.3) than in the non-converter groups (mean duration = 25.8 weeks, SD = 28.7) (*t* = 4.926, *p* < 0.001).

### Measurement of the complement and inflammatory levels

Venous blood samples were collected at baseline. All samples were drawn in the morning, and the participants fasted for a minimum of 3 h prior to blood collection. Ten mL of peripheral venous blood was drawn into anticoagulant-free tubes. The samples were kept at room temperature for 1 h, followed by centrifugation (1710 *g* for 20 min at 4 °C) for serum separation. Subsequently, the serum was separated and stored at −80 °C until analysis.

Twelve complement proteins (C1q, C2, C3, C3b, C4, C4b, C5, C5a, factor B, D, I, H) from the MILLIPLEX MAP Human Complement Magnetic Bead Panel 2 - Immunology Multiplex Assay (HCMP2MAG-19K, Merck Millipore, Billerica, MA, USA) were quantified in duplicate using the Luminex 200 system (Luminex Corporation, Austin, TX, USA). Two duplicate tests were conducted on the standard and reference samples. The participants’ samples were measured in singlets. The coefficient of Variance of all standards and reference materials were less than 20%. According to the fluorescence detection value obtained from the standard product, the standard curve was fitted using the multiparameter mode to obtain the standard curve and its equation. The concentration units were expressed as picograms of protein per milliliter of serum (pg/mL). In the standard curve, the ratio of the detected value (observed concentration, Obs) to the expected value (expected concentration, Exp) reflects the quality of the standard curve. More than 97% of the standards (Obs/Exp) × 100 were between 70% and 130%, indicating that the standard curve works well. Inflammatory and complement concentrations that were too low and lower than the detection range of the standard curve (LQ) were not included in the analysis. Only one CHR sample had a complement concentration value of LQ, six CHR samples with LQ had an inflammatory concentration value of LQ, and four HC samples with LQ had an inflammatory concentration value of LQ. The Chi-square tests were not significant when the number of LQ between the CHR and HC groups and the conversion and non-conversion groups were compared. A summary of the quality control information with the coefficient of variation details is presented in Supplementary 2.

The serum levels of macrophage colony-stimulating factor (GM-CSF), interleukin (IL)-10(IL-10), IL-1beta, IL-6, IL-8, and tumor necrosis factor-alpha (TNF-α) were measured for each sample in duplicate using an enzyme-linked immunosorbent assay with the Human HS Cytokine Premixed Kit (catalog #: FCSTM09-10, USA) according to the manufacturer’s instructions. The concentration of inflammatory cytokines was expressed in pg/mL. All data were calibrated using standard curves generated for each cytokine. Inflammatory concentration values that were too low and below the detection range of the standard curve were not included in the main statistical analysis.

### Data analysis

The sociodemographic and clinical characteristics of individuals at CHR and HC are presented as descriptive statistics, such as percentages and mean scores (standard deviation, S.D.). The CHR individuals were classified into converter and non-converter groups according to the 1-year follow-up assessment results. Demographic and clinical characteristics were presented, and variables were compared across groups using independent-sample *t*-tests (for continuous variables) or chi-square tests (for categorical variables). The normality of the distribution of complement and inflammatory variables was assessed using the Kolmogorov-Smirnov test; however, none were normally distributed. Comparisons were performed using non-parametric analyses. Spearman’s rank correlations were calculated between the clinical variables and complement or inflammatory levels. Linear regression models were used to explore the relationship between symptom/function changes (follow-up value minus baseline value) and inflammatory/complement factors. A bar plot diagram was created using GraphPad Prism software to analyze the differences in the serum levels of complement and inflammatory variables between CHR vs. HC and converter vs. non-converter. The predictive performance of the complement and inflammatory variables in discriminating converters from non-converters was evaluated using receiver operating characteristic (ROC) curve analysis. The discriminative ability of complement and inflammation was evaluated using the area under the ROC curve (ROC–AUC) for the conversion outcome. According to an optional classification, the AUC can be defined as follows: 0.9–1, excellent; 0.8–0.9, good; 0.7–0.8, fairly good; 0.6–0.7, weak; and 0.5–0.6, useless [37]. Finally, logistic regression was used to calculate the odds ratio (OR) and 95% confidence interval (CI) for the association between the individual complement or inflammatory level and conversion outcomes.

## Results

### Baseline demographics and clinical characteristics

The baseline characteristics of the participants are summarized in Table 1. No significant differences in the baseline demographic characteristics and baseline clinical characteristics were identified between the CHR and HC groups. Clinical data from the 3-year follow-up are presented in Table S2.

**Table 1:** Baseline demographic and clinical variables, comparison between CHR and HC, CHR-converter and CHR-non-converter.

Variables	CHR- total	HC	Comparison		CHR-converter	CHR-non-converter	Comparison	
			*t/χ*^*2*^	*P*			*t/χ*^*2*^	*P* value
Cases (*n*)	49	26	-	-	25	24	-	-
Demographic variables [mean (S.D.)]								
Age (years)	18.2(4.1)	17.4(2.3)	0.967	0.337	18.1(4.1)	18.4(4.2)	0.251	0.803
Male [*n*(%)]	35(71.4)	18(69.2)	*χ*^*2*^ = 0.040	0.842	18(72.0)	17(70.8)	*χ*^*2*^ = 0.008	0.928
Female [*n*(%)]	14(28.6)	8(30.8)			7(28.0)	7(29.2)		
Education (years)	10.4(2.7)	11.2(2.0)	1.304	0.196	10.2(2.2)	10.5(3.2)	0.441	0.661
Weight (kg)	62.8(12.8)	61.5(13.9)	0.411	0.683	62.0(14.1)	63.5(11.5)	0.407	0.686
Height (cm)	167.4(7.9)	168.7(9.4)	0.646	0.520	167.0(7.1)	167.9(8.8)	0.403	0.689
BMI	22.4(4.3)	21.5(4.2)	0.840	0.404	22.3(5.0)	22.5(3.6)	0.193	0.848
History(none), [*n*(%)]	39(79.6)	-	-	-	22(88.0)	17(70.8)	*χ*^*2*^ = 4.527	0.104
History(low-risk), [*n*(%)]	7(14.3)	-	-	-	1(4.0)	6(25.0)		
History(High-risk), [*n*(%)]	3(6.1)	-	-	-	2(8.0)	1(4.2)		
SIPS variables [Mean (S.D.)]								
APSS, [*n*(%)]	45(91.8)	-	-	-	23(92.0)	22(91.7)	*χ*^*2*^ = 0.356	0.837
GRDS, [*n*(%)]	3(6.1)	-	-	-	1(4.0)	2(8.3)		
BIPS, [*n*(%)]	4(8.2)	-	-	-	2(8.0)	2(8.3)		
Before GAF	77.1(4.3)	-	-	-	76.9(4.2)	77.4(4.6)	0.365	0.717
Now GAF	52.0(8.6)	-	-	-	52.2(7.5)	51.7(9.7)	0.199	0.843
GAF drop	25.2(7.5)	-	-	-	24.7(7.5)	25.7(7.7)	0.435	0.665
Positive symptoms	10.1(4.0)	-	-	-	10.3(3.6)	9.8(4.5)	0.423	0.674
Negative symptoms	13.8(6.6)	-	-	-	13.7(6.8)	13.9(6.5)	0.082	0.935
Disorganization symptoms	6.9(3.4)	-	-	-	6.7(2.8)	7.1(3.9)	0.417	0.678
General symptoms	8.9(3.3)	-	-	-	8.8(3.0)	8.9(3.6)	0.124	0.902
SOPSTAL	39.7(11.3)	-	-	-	39.6(10.2)	39.8(12.5)	0.058	0.954

*BMI* body mass index, *GAF drop* GAF (Global Assessment of Functioning) score baseline from highest in the past year, *low-risk family history* having any family members with mental disorders or a first-degree relative with non-psychotic disorders; *high-risk family history* having at least one first-degree relative with psychosis, *APSS* attenuated positive symptom syndrome, *GRDS* genetic risk and deterioration syndrome, *BIPS* brief intermittent psychotic syndrome, *CHR* Clinical high risk for psychosis, *CHR-converter* CHR individuals who were converted to full psychosis, *HC* Healthy control, *t/χ*^*2*^ *t* for independent *t* test, *χ*^*2*^ for kappa test.

### Complement/inflammatory factors between CHR individuals and HC as well as converters and non-converters

**Fig. 2:**
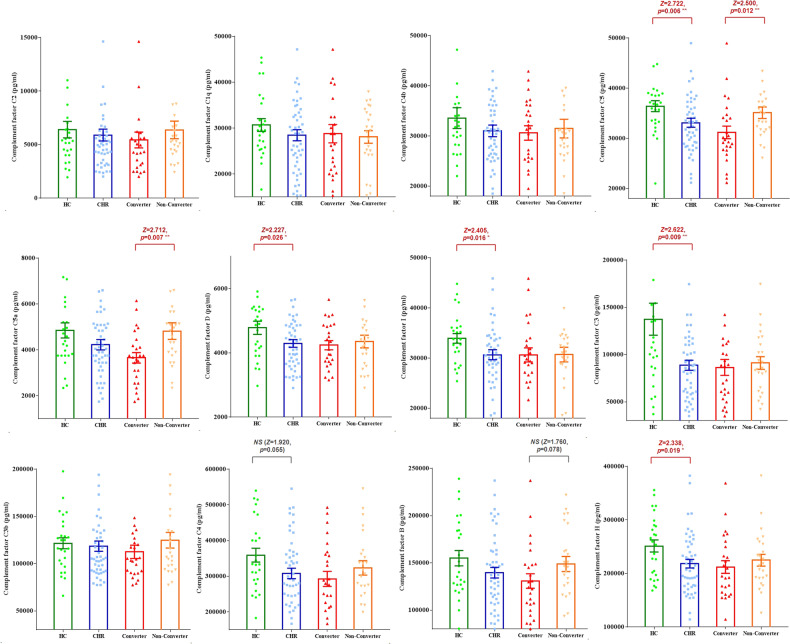
Comparisons for serum levels of complement factors in HC and CHR individuals with converter versus non-converter. Bar shows the mean and standard error of complement factors. The Mann-Whitney *U* tests were performed for comparisons between groups. The level of statistical significance was set at a two-tailed P value of 0.05. Statistically significant p values are written above the bars. NS: non statistic significant. CHR Clinical high risk for psychosis, CHR-converter, CHR individuals who were converted to fully psychosis; HC Healthy control, ^*^p < 0.05; ^**^p < 0.01; ^***^p < 0.001.

We compared the levels of complement factors between HC and CHR individuals with the conversion and non-conversion outcomes (Fig. 2). The C5 (CHR, mean = 33103.3 pg/mL vs. HC, mean = 36413.5 pg/mL), C3 (CHR, mean = 88664.8 pg/mL vs. HC, mean = 137386.7 pg/mL), D (CHR, mean = 4293.9 pg/mL vs. HC, mean = 4779.4 pg/mL), I (CHR, mean = 30662.3 pg/mL vs. HC, mean = 33921.7 pg/mL), and H (CHR, mean = 218174.3 pg/mL vs. HC, mean = 251216.3 pg/mL) levels were significantly lower in the CHR group than in the HC group (C5, *z* = 2.722, *p* = 0.006; C3, *z* = 2.622, *p* = 0.009; D, *z* = 2.227, *p* = 0.026; I, *z* = 2.405, *p* = 0.016; H, *z* = 2.338, *p* = 0.019). Compared to non-converters, converters had significantly lower levels of C5 (CHR-converter, mean = 31177.5 pg/mL vs. non-converter, mean = 35109.4 pg/mL, *z* = 2.500, *p* = 0.012) and C5a (CHR-converter, mean = 3650.8 pg/mL vs. non-converter, mean = 4819.4 pg/mL, *z* = 2.712, *p* = 0.007). Except for the higher level of TNF-alpha in HC compared to CHR, none of the inflammatory factors reached a significant level in either the comparison of CHR individuals and HC or between CHR-converters and non-converters (Fig. S1).

### Correlation between functional and symptomatic changes and complement/inflammatory factors

**Fig. 3:**
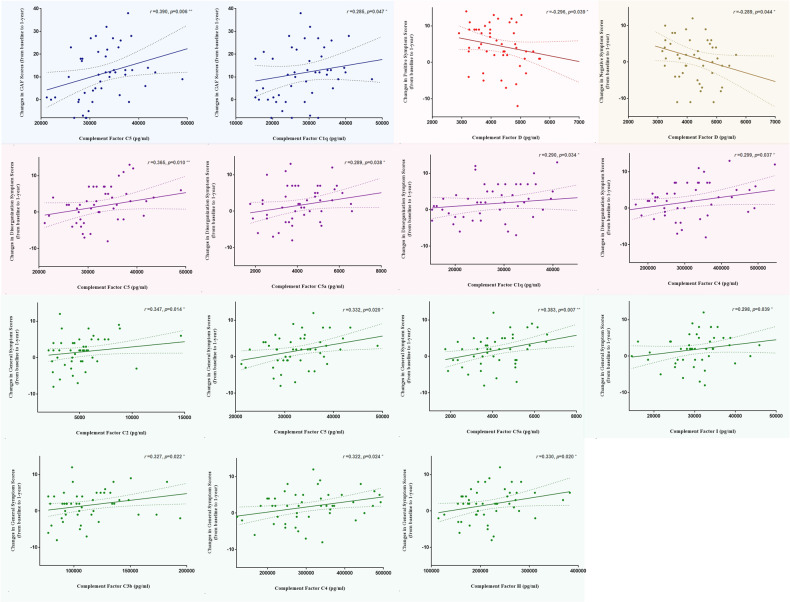
Correlation analysis between functional and symptomatic changes and complement factors. Correlation (Spearman) between functional and symptomatic changes from baseline to 1-year and complement factors. GAF Global Assessment of Functioning, ^*^p < 0.05; ^**^p < 0.01; ^***^p < 0.001.

As shown in Fig. 3, there were significant correlations between the changes in the GAF score and C5, C1q; changes in the score of positive symptoms and factor D; changes in the score of negative symptoms and factor D; changes in the score of disorganization symptoms and C5, C5a, C1q, and C4; and changes in the score of general symptoms and C2, C5, C5a, factor I, C3b, C4, and factor H (Table S3). None of the inflammatory factors showed a significant correlation between changes in general function and symptoms (Table S4). Using linear regression, there was no statistically significant relationship between functional and symptomatic changes and inflammatory factors (none of the factors were entered into the model) when controlled for multiple correlations. However, there was a statistically significant relationship between complement H and C5 levels and symptomatic changes (Table S5). In order to avoid the potential impact of baseline symptom differences in correlation analysis, further analysis was applied by using the follow-up functional and symptomatic values as the dependent variables, the baseline functional and symptomatic values as a covariate, complement/inflammatory factors as the independent variables in the linear regression analysis, results are similar (Table S5).

### Discrimination of the CHR state and conversion outcome

**Fig. 4:**
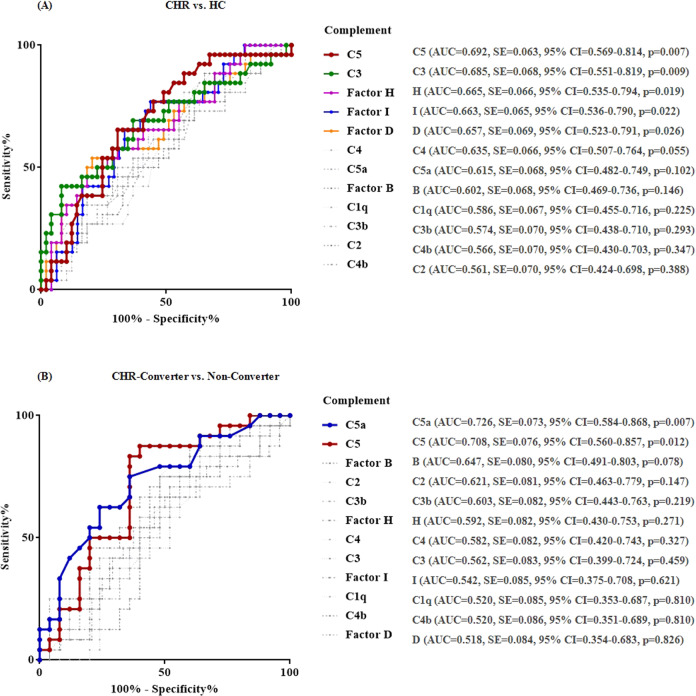
Receiver operating characteristic curve profiles for complement factors in terms of discrimination of the CHR individuals from HC, and CHR non-converters from converters. Abbreviations: AUC an area under the Receiver operating characteristic curve, CHR Clinical high risk for psychosis, CHR-converter, CHR individuals who were converted to fully psychosis, HC Healthy control.

The ROC analysis for each complement factor resulted in an AUC ranging from 0.657 to 0.692 for C5, C3, factors H, I, and D, which were significant in the discrimination of the CHR state from HC, and an AUC of 0.726 for C5a (SE [standard error] = 0.073, *p* = 0.007, 95% CI [confidence interval] = 0.584–0.868, Cut-off value = 4251, Sensitivity = 62.5%, Specificity = 76.0%, Likelihood ratio = 2.604) and 0.708 for C5 (SE = 0.076, *p* = 0.012, 95% CI = 0.560-0.857, Cut-off value = 31169, Sensitivity = 83.3%, Specificity = 64.0%, Likelihood ratio = 2.315), which were significant in the discrimination of conversion outcome in CHR individuals (Fig. 4). Except for the TNF-α level in the discrimination of CHR individuals and HC, none of the inflammatory factors reached a significant level in the ROC analysis of discrimination between CHR individuals and HC, CHR-converter, and CHR-non-converter (Fig. S3).

## Discussion

### Key findings

Although both inflammatory and complement factors have been reported to be significantly different between CHR individuals and HC [3, 18], very few studies have been conducted to compare the two types of biomarkers in predicting psychosis. To our knowledge, this study is the first to directly compare the predictive performance of a wide range of inflammatory and complement factors between CHR groups with converter and non-converter outcomes. Compared to the insignificant difference found in the inflammatory factors, the complement factors showed more significant differences between CHR individuals and HC, and lower levels of C5 and C5a in CHR individuals were associated with a higher risk of conversion to psychosis. This study provides clear evidence that measuring the levels of complement factors is more effective than applying inflammatory factors in predicting conversion to psychosis in the CHR population.

### CHR versus HC subjects

The comparison of cytokine and complement factors between CHR individuals and HC reflected the immuno-inflammatory imbalance characteristics of individuals at CHR. Among the inflammatory factors, only the level of TNF-α was significantly lower in the CHR group than in the HC group. Karanikas et al. [38, 39]. reported lower serum levels of TNF-α, IL-2, and IL-10 in 12 males at CHR than in 25 males with first-episode psychosis. We failed to find significant differences in most cytokine levels between the CHR and HC groups. In contrast, the complement factors C5, C3, D, I, and H were significantly lower in the CHR group than in the HC group. Similarly, Idonije et al. [17]. found that complement C1q and C3c levels were significantly reduced in newly diagnosed patients with schizophrenia compared to controls. Several previous studies [28, 40–42] have measured peripheral inflammatory cytokines and complement proteins in psychosis, producing mixed results. Our finding of decreased TNF-α and some complement factors in CHR populations is in contrast to studies reporting increased immuno-inflammatory levels in patients with established psychosis [3, 4, 43, 44]. The reason for these discrepancies is unclear but may be related to the fact that immune activation may occur at an earlier stage in individuals at CHR who would convert to psychosis, such as in the pre-morbid phase; overconsumption drives subsequent decreases in the CHR phase. Other explanations include variations in measurement techniques, confounders of antipsychotic medication in patients with psychosis, the stage of illness, and sample heterogeneity. A recent systematic review [45] suggested that antipsychotics have a range of immunomodulatory effects, including acting upon cytokine networks [46] which may cause those patients with psychosis who had taken antipsychotic drugs to have significantly higher levels of complement factors than drug-naïve patients. The current CHR population is drug-naive. Therefore, the low complement levels found in this study may be closer to the real situation of complement levels at the risk stage of psychosis.

### Converter versus non-converter

Similarly, there was no statistically significant difference in inflammatory cytokines between the CHR-converter and non-converter groups. No significant correlation was found between cytokine levels and changes in general function or symptoms during the follow-up. However, complement factors, specifically C5 and C5a, have shown significantly lower levels in converters than in non-converters. The C5 and C5a levels in the non-converter group were similar to those in the HC group. Furthermore, many complement factors are significantly correlated with changes in general function and symptoms. The main reason for the difference in the complement system between CHR-converters and non-converters being more obvious than that of inflammatory factors may be that the complement system plays multiple roles in both defense responses against infection and inflammatory pathology [47]. Both microbial components, inflammatory cytokines, and immune complexes can activate complement factors.

Dysregulation of the complement system may also occur earlier than an inflammatory imbalance in the development of psychosis and thus is not detectable for the difference in inflammatory factors in the CHR population. Supporting evidence from prospective studies [48, 49] has reported that the complement system is dysregulated in the blood during childhood before the development of psychosis. They found that the complement cascade related to 60 of 181 plasma proteins was differentially expressed in children with psychosis. There is also evidence [50] that these complement disturbances may even span generations, with one study finding that higher maternal complement C1q was associated with increased odds of psychosis in adult offspring compared with mothers whose children did not develop mental disorders.

### Complement versus inflammatory in prediction

Consistently, in the inflammation-specific prediction model, none of the cytokines contributed significantly to the prediction of conversion. However, in the complement-specific prediction model, C5 and C5a significantly contributed to the prediction of conversion. In the few studies on the complement levels of patients with psychosis, most of the results focused on complement C3 and C4 [12], while there are few studies on the role of complement C5 in psychosis, and most of them are insignificant findings [4, 17]. In general, complement activation follows various pathways, and the final step of the pathway converges upon the cleavage of complement C5 into complement fragments C5a and C5b. Considering that the complement system plays a role in synapse elimination/pruning, which may be involved in the pathogenesis of psychosis, C5 levels may be altered during the development of psychosis. The findings of these studies support this hypothesis. Allswede et al. [51]. identified that the peripheral mRNA expression level of the complement gene (C5) made unique contributions to the variance in superior frontal cortical thickness. Ishii et al. [52]. found elevated C5 levels in the cerebrospinal fluid of patients with schizophrenia.

### Limitations

Despite the current study measuring a relatively wide range of inflammatory cytokines and complement factors in a clinical population at the CHR stage with a longitudinal follow-up to determine the outcomes, the present study has several limitations. First, the sample size was small and potentially inadequately powered, which could have introduced sampling errors and increased the chance of type II errors in the analysis. Second, the current study had a high drop-out rate (102/151 dropped), which may have resulted in a higher conversion rate in this sample than in other CHR studies. Due to COVID-19-related movement restrictions, most CHR individuals who had been alleviated no longer went to the hospital, and only those patients with progressive disease still presented. As a result, most participants who completed the face-to-face follow-ups were more serious patients with progressive disease (converted to psychosis, for example). Specifically, CHR individuals included in this study have selective bias, that is, they show more severe negative symptoms and impaired functions at baseline. Therefore, we need to be cautious when applying the conclusions of this study, especially in CHR populations with less symptoms and lower risk. Third, complement factors were measured in peripheral serum. However, it remains unclear how peripheral levels of complement are related to complement in the brain. Fourth, although most CHR individuals were converted within 3 years after enrollment, some were converted after 3 years, which may have led to an underestimation of the number of CHR converters.

## Conclusion

In summary, this study further demonstrates that the serum levels of complement in individuals at CHR are more strongly associated with conversion to psychosis than inflammatory factors. Therefore, alterations in the serum complement levels may precede the first episode of psychosis in individuals at CHR. Complement may also be important for developing specific strategies to monitor and treat early psychosis. The time sequence, interaction, and causal relationships between complement proteins and inflammatory cytokines require further clarification.

## Supplementary information

Supplementary 1

Supplementary 2
