# Supplementary material for: Serum complement proteins rather than inflammatory factors is effective in predicting psychosis in individuals at clinical high risk
Source: Transl Psychiatry. 2023 Jan 12;13:9. doi: 10.1038/s41398-022-02305-1 (PMC9834035; doi:10.1038/s41398-022-02305-1)
Supplement: Supplementary file 1 — Supplementary 1 [file 41398_2022_2305_MOESM1_ESM.docx]

**Supplementary**

**Table s1**. Baseline demographic, clinical and cytokine variables, comparison of participants included for analysis and excluded during the same period.

| Variables | CHR- included | CHR- excluded | Comparison | |
| --- | --- | --- | --- | --- |
|  |  |  | *t/****χ^2^*** | *P* value |
| Cases(n) | 49 | 102 | - | - |
| Age(years)[mean(S.D.)] | 18.2 (4.1) | 18.3(4.6) | 0.039 | 0.969 |
| Male[n(%)] | 35(71.4) | 45(44.1) | ***χ^2^***=9.911 | **0.002** |
| Female[n(%)] | 14(28.6) | 57(55.9) |  |  |
| Education(years)[mean(S.D.)] | 10.4(2.7) | 10.8(2.9) | 0.765 | 0.446 |
| Family history(none)[n(%)] | 39(79.6) | 83(81.4) | ***χ^2^***=0.208 | 0.901 |
| Family history(low-risk),[n(%)] | 7(14.3) | 12(11.8) |  |  |
| Family history(High-risk),[n(%)] | 3(6.1) | 7(6.9) |  |  |
| APSS,[n(%)] | 45(91.8) | 97(95.1) | ***χ^2^***=1.117 | 0.572 |
| GRDS,[n(%)] | 3(6.1) | 3(2.9) |  |  |
| BIPS,[n(%)] | 4(8.2) | 6(5.9) |  |  |
| Before GAF[mean(S.D.)] | 77.1(4.3) | 78.1(4.7) | 1.133 | 0.259 |
| Current GAF[mean(S.D.)] | 52.0(8.6) | 55.7(7.2) | 2.809 | **0.006** |
| GAF drop[mean(S.D.)] | 25.2(7.5) | 22.3(6.8) | 2.316 | **0.022** |
| Positive symptoms [Mean(S.D.)] | 10.1(4.0) | 10.1(3.6) | 0.055 | 0.956 |
| Negative symptoms [Mean(S.D.)] | 13.8(6.6) | 11.4(5.8) | 2.320 | **0.022** |
| Disorganization symptoms [Mean(S.D.)] | 6.9(3.4) | 6.4(3.0) | 1.003 | 0.318 |
| General symptoms [Mean(S.D.)] | 8.9(3.3) | 9.5(2.7) | 1.203 | 0.231 |
| Total score [Mean(S.D.)] | 39.7(11.3) | 37.3(10.6) | 1.241 | 0.217 |

**Note.** GAF drop, GAF (Global Assessment of Functioning) score baseline from highest in the past year; low-risk family history, having any family members with mental disorders or a first-degree relative with non-psychotic disorders; high-risk family history, having at least one first-degree relative with psychosis; APSS, attenuated positive symptom syndrome; GRDS, genetic risk and deterioration syndrome; BIPS, brief intermittent psychotic syndrome; CHR, Clinical high risk for psychosis; *t/χ^2^*: *t* for independent t test, *χ^2^* for kappa test. The level of statistical significance was set at a P value of 0.05. Bold indicates significant values.

**Table s2**. Follow-up clinical variables, comparison of CHR-converter and CHR-non-converter.

| Variables | CHR-converter | CHR-non-converter | Comparison | |
| --- | --- | --- | --- | --- |
|  |  |  | *t* | *P* value |
| Cases(n) | 25 | 24 | - | - |
| SIPS variables at 1 year later | | | | |
| GAF at 1-year [Mean(S.D.)] | 59.2(12.3) | 68.5(9.4) | 2.993 | **0.004** |
| Positive symptoms [Mean(S.D.)] | 8.0(5.2) | 3.0(2.1) | 4.345 | **<0.001** |
| Negative symptoms [Mean(S.D.)] | 14.6(7.5) | 10.8(6.2) | 1.929 | 0.060 |
| Disorganization symptoms [Mean(S.D.)] | 6.6(5.2) | 3.8(2.8) | 2.377 | **0.023** |
| General symptoms [Mean(S.D.)] | 8.1(2.4) | 6.0(3.1) | 2.686 | **0.010** |
| SOPSTAL [Mean(S.D.)] | 37.3(17.1) | 23.5(12.0) | 3.245 | **0.002** |

**Note.** CHR, Clinical high risk for psychosis; CHR-converter, CHR individuals who were converted to fully psychosis; GAF, Global Assessment of Functioning; *t* for independent t test. The level of statistical significance was set at a P value of 0.05. Bold indicates significant values.

**Table-s3.** Correlation analysis between functional and symptomatic changes and complement factors.

| Changes | | C1q | C2 | C3 | C3b | C4 | C5 | C5a | B | D | I | H |
| --- | --- | --- | --- | --- | --- | --- | --- | --- | --- | --- | --- | --- |
| Positive Symptoms | *r* | .004 | -.117 | -.081 | -.127 | .002 | .159 | .237 | -.026 | -.296* | -.023 | -.130 |
|  | *p* | .980 | .422 | .579 | .383 | .991 | .275 | .102 | .860 | .039 | .877 | .373 |
| Negative Symptoms | *r* | .042 | -.092 | .065 | -.158 | .207 | .215 | .165 | .159 | -.289* | .104 | .012 |
|  | *p* | .772 | .528 | .658 | .279 | .154 | .139 | .258 | .275 | .044 | .483 | .935 |
| Disorganization Symptoms | *r* | .290* | -.015 | .106 | .099 | .299* | .365** | .298* | .276 | -.233 | .114 | .106 |
|  | *p* | .043 | .917 | .469 | .501 | .037 | .010 | .038 | .055 | .107 | .439 | .470 |
| General Symptoms | *r* | .273 | .347* | .148 | .327* | .322* | .332* | .383** | .281 | .048 | .298* | .330* |
|  | *p* | .058 | .014 | .310 | .022 | .024 | .020 | .007 | .051 | .743 | .039 | .020 |
| GAF scores | *r* | .285* | -.004 | .242 | .025 | .269 | .390** | .255 | .255 | -.159 | .163 | .165 |
|  | *p* | .047 | .979 | .093 | .863 | .062 | .006 | .077 | .077 | .276 | .268 | .257 |

**Note.** Correlation (Spearman) between functional and symptomatic changes from baseline to 1-year and complement factors. GAF, Global Assessment of Functioning CHR, Clinical high risk for psychosis; Spearman correlation r, correlation coefficient; *The level of statistical significance was set at a P value of 0.05.

**Table-s4.** Correlation analysis between functional and symptomatic changes and inflammatory factors

| Changes | | | GM-CSF | | IL-10 | | IL-1beta | IL-6 | | IL-8 | | TNF-alpha |
| --- | --- | --- | --- | --- | --- | --- | --- | --- | --- | --- | --- | --- |
| Positive Symptoms | *r* | -.197 | | .002 | | .063 | | .072 | -.241 | | -.203 | |
|  | *p* | .175 | | .990 | | .700 | | .633 | .095 | | .162 | |
| Negative Symptoms | *r* | .006 | | -.047 | | -.068 | | .071 | -.244 | | -.130 | |
|  | *p* | .965 | | .799 | | .676 | | .640 | .092 | | .372 | |
| Disorganization Symptoms | *r* | -.044 | | -.183 | | .110 | | .089 | -.165 | | -.053 | |
|  | *p* | .764 | | .315 | | .501 | | .558 | .258 | | .716 | |
| General Symptoms | *r* | -.164 | | .236 | | -.087 | | -.068 | .082 | | -.055 | |
|  | *p* | .259 | | .194 | | .592 | | .652 | .576 | | .708 | |
| GAF scores | *r* | -.113 | | -.019 | | .069 | | .098 | -.068 | | -.140 | |
|  | *p* | .439 | | .918 | | .673 | | .516 | .643 | | .338 | |

**Note.** Correlation (Spearman) between functional and symptomatic changes from baseline to 1-year and inflammatory factors. GAF, Global Assessment of Functioning CHR, Clinical high risk for psychosis; GM-CSF, macrophage colony-stimulating factor; IL-10, interleukin (IL)-10; IL-1beta, interleukin (IL)-1beta; IL-6, interleukin (IL)-6; IL-8, interleukin (IL)-8; TNF-alpha, tumor necrosis factor-alpha. Spearman correlation r, correlation coefficient.

**Table s5.** Linear regression of functional and symptomatic changes as dependent value from predictor inflammatory/complement factors.

| (Dependent variable) Changes in Positive Symptoms |
| --- |
| Inflammatory factors (None of factor was significant) (p value ranged from 0.474-0.950) |
| Complement factors (Complement C5 (t=2.269, p=0.029) and H (t=2.134, p=0.040) were significant in the Linear regression) |
| (Dependent variable) Follow-up Positive Symptoms, (Covariate) Baseline Positive Symptoms |
| Inflammatory factors (None of factor was significant) (p value ranged from 0.625-0.994) |
| Complement factors (Complement C2 (t=2.159, p=0.038), Complement C5 (t=-2.154, p=0.038) were significant) and (Complement H (t=1.929, p=0.062) was trend to be significant) in the model |
| (Dependent variable) Changes in Negative Symptoms |
| Inflammatory factors (None of factor was significant) (p value ranged from 0.276-0.688) |
| Complement factors (None of factor was significant) (p value ranged from 0.112-0.843) |
| (Dependent variable) Follow-up Negative Symptoms, (Covariate) Baseline Negative Symptoms |
| Inflammatory factors (None of factor was significant) (p value ranged from 0.300-0.798) |
| Complement factors (None of factor was significant) (p value ranged from 0.140-0.825) |
| (Dependent variable) Changes in Disorganization Symptoms |
| Inflammatory factors (None of factor was significant) (p value ranged from 0.566-0.926) |
| Complement factors (Complement H (t=2.543, p=0.015) was significant and Complement C5 (t=1.839, p=0.074), Complement D (t=1.801, p=0.080) were trend to be significant in the Linear regression |
| (Dependent variable) Follow-up Disorganization Symptoms, (Covariate) Baseline Disorganization Symptoms |
| Inflammatory factors (None of factor was significant) (p value ranged from 0.408-0.886) |
| Complement factors (None of factor was significant) (p value ranged from 0.133-0.922) |
| (Dependent variable) Changes in General Symptoms |
| Inflammatory factors (None of factor was significant) (p value ranged from 0.225-0.885) |
| Complement factors (None of factor was significant) (p value ranged from 0.214-0.960) |
| (Dependent variable) Follow-up General Symptoms, (Covariate) Baseline General Symptoms |
| Inflammatory factors (None of factor was significant) (p value ranged from 0.154-0.901) |
| Complement factors (None of factor was significant) (p value ranged from 0.166-0.981) |
| (Dependent variable) Changes in GAF scores |
| Inflammatory factors (None of factor was significant) (p value ranged from 0.242-0.958) |
| Complement factors (Complement H (t=1.959, p=0.058) and Complement C5 (t=1.863, p=0.071) were trend to be significant in the model |
| (Dependent variable) Follow-up GAF scores, (Covariate) Baseline GAF scores |
| Inflammatory factors (None of factor was significant) (p value ranged from 0.190-0.911) |
| Complement factors Complement C5 (t=2.081, p=0.045) was significant in the model. |

Notes: GAF, Global Assessment of Functioning. Bate is the regression coefficient. SE is the standard error. 95% CI is the estimated 95% confidence interval for the corresponding parameter. β(OR) is the standardized regression coefficient. Bold in significant.

**Figure s1.** Comparisons for serum levels of inflammatory factors in HC and CHR individuals with converter versus non-converter


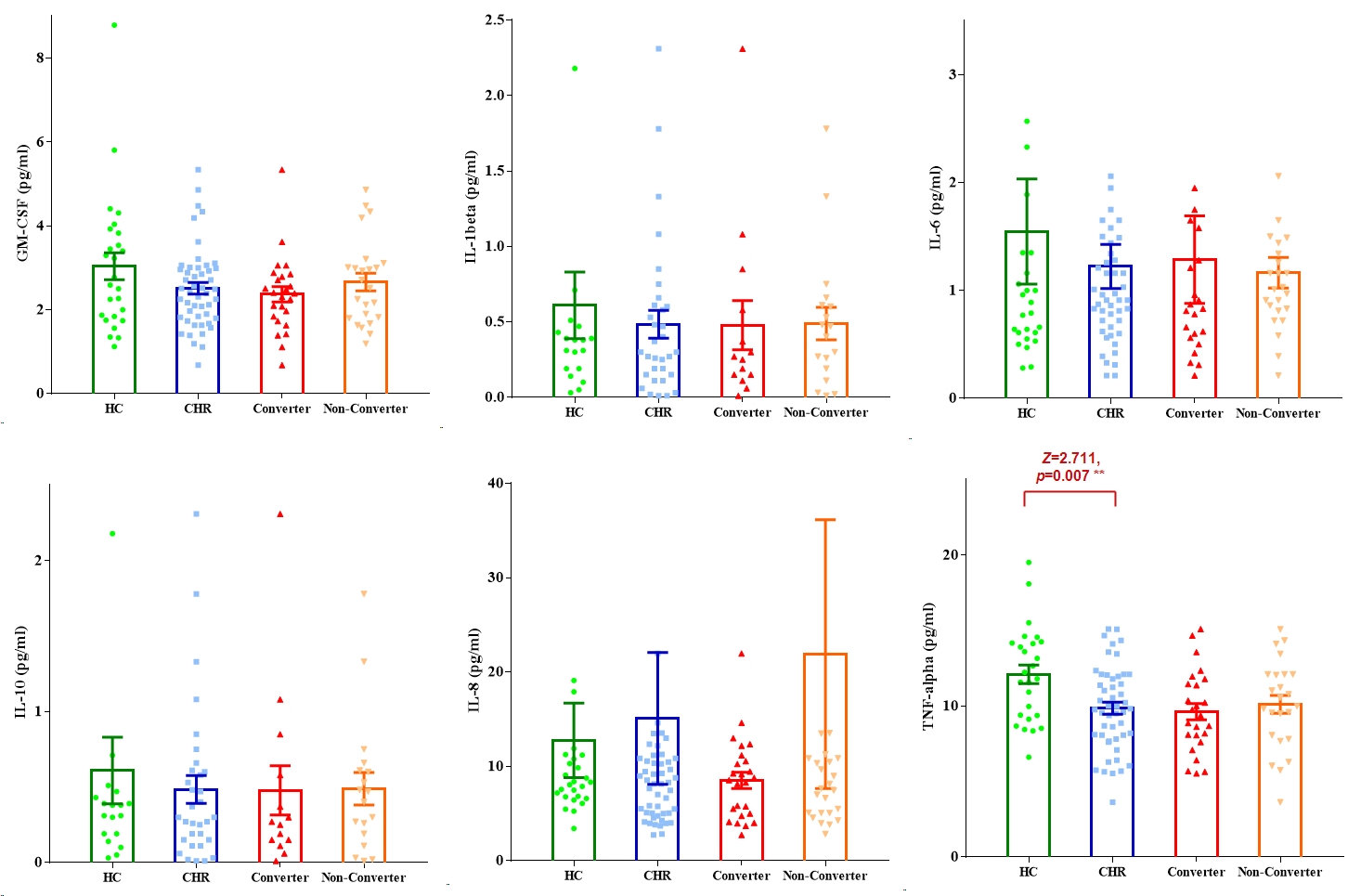


**Note.** Bar shows the mean and standard error of inflammatory factors. The Mann-Whitney U tests were performed for comparisons between groups. The level of statistical significance was set at a two-tailed P value of 0.05. Statistically significant p values are written above the bars. GM-CSF, macrophage colony-stimulating factor; IL-10, interleukin (IL)-10; IL-1beta, interleukin (IL)-1beta; IL-6, interleukin (IL)-6; IL-8, interleukin (IL)-8; TNF-alpha, tumor necrosis factor-alpha. CHR, Clinical high risk for psychosis; CHR-converter, CHR individuals who were converted to fully psychosis; HC, Healthy control; ^*^*p* <0.05; ^**^*p* < 0.01; ^***^*p* < 0.001.

**Figure s2.** Receiver operating characteristic curve profiles for inflammatory factors in terms of discrimination of the CHR individuals from HC, and CHR non-converters from converters.


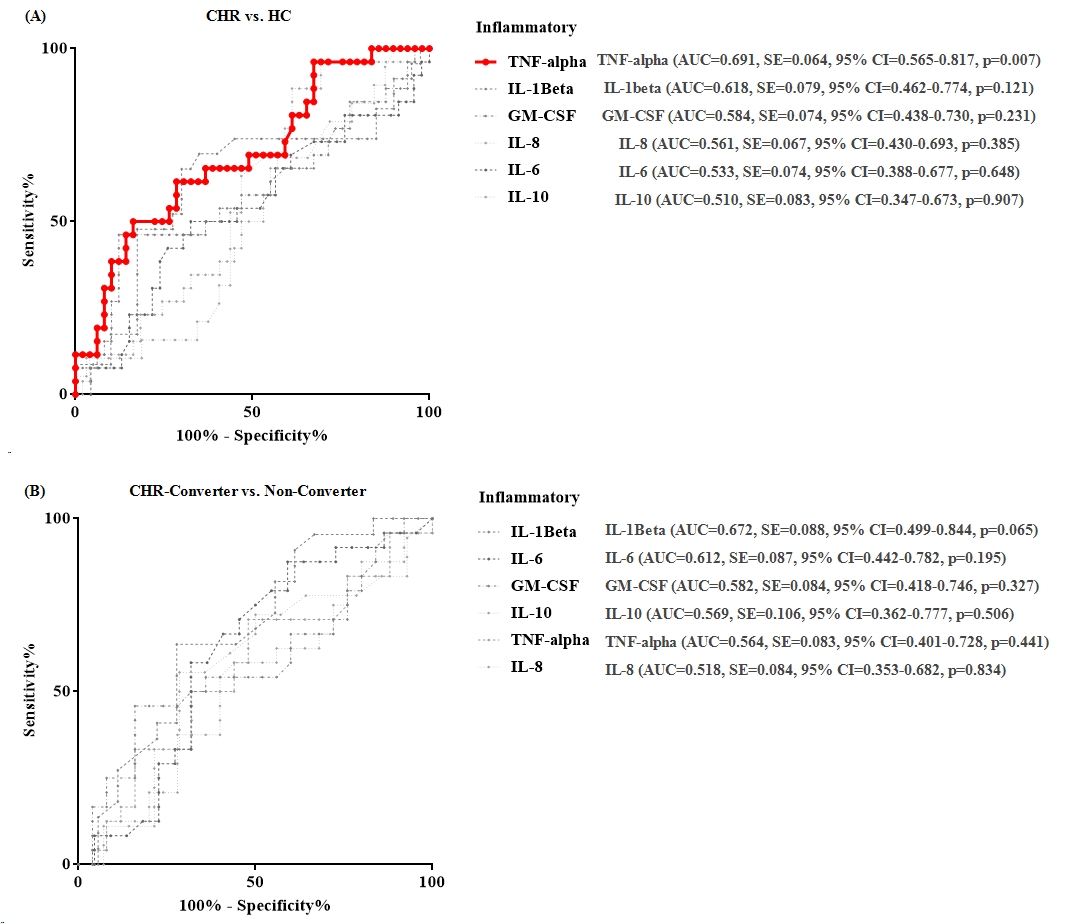


**Note.** Abbreviations: AUC, an area under the Receiver operating characteristic curve; GM-CSF, macrophage colony-stimulating factor; IL-10, interleukin (IL)-10; IL-1beta, interleukin (IL)-1beta; IL-6, interleukin (IL)-6; IL-8, interleukin (IL)-8; TNF-alpha, tumor necrosis factor-alpha. CHR, Clinical high risk for psychosis; CHR-converter, CHR individuals who were converted to fully psychosis; HC, Healthy control.
