# Supplementary material for: Serum complement proteins rather than inflammatory factors is effective in predicting psychosis in individuals at clinical high risk
Source: Transl Psychiatry. 2023 Jan 12;13:9. doi: 10.1038/s41398-022-02305-1 (PMC9834035; doi:10.1038/s41398-022-02305-1)
Supplement: Supplementary file 2 — Supplementary 2 [file 41398_2022_2305_MOESM2_ESM.pdf]

ID	Type	Well	Complement.C1q.	Complement.C2	Complement.C3.
S1	S1	E2,F2	1.84	3.45	3.91
S2	S2	C2,D2	6.26	14.43	4.02
S3	S3	A2,B2	4.92	3.82	8.98
S4	S4	G1,H1	4.38	N/A	6.46
S5	S5	E1,F1	6.28	2.32	2.16
S6	S6	C1,D1	5.92	0.97	3.79
S7	S7	A1,B1	0.85	0.74	7.39
C1	C1	A3,B3	4.49	0.78	0.93
C2	C2	C3,D3	2.71	1.25	1.22

S: Standard
C: Reference

Complement.C3b	Complement.C4.	Complement.C4b	Complement.C5	Complement.C5a
0.91	1.94	5.66	N/A	4.29
1.42	6.82	N/A	12.86	9.43
2.47	1.42	4.12	N/A	3.1
3.26	4.38	4.94	1.17	0
0.69	7.38	0.42	1.7	1.18
0.2	11.24	0.66	0.5	0
1.14	6.82	0.08	2.17	1.08
0.53	5.15	4.27	6.96	1.68
1.04	4.93	0.1	5.62	1.13

Complement.Factor.B.	Complement.Factor.D.	Complement.Factor.I.	Complement.Factor.H.
5.98	N/A	6.15	8.76
5.76	2.57	5.44	8.45
4.11	4.71	1.48	6.3
4.69	3.26	1.07	5.36
5.02	1.84	2.32	4.9
4.1	0.67	2.25	8.49
0.8	0.65	0.28	2.05
1.64	0.71	2.68	1.4
3.62	0.77	0.86	0.27

ID	Type	Well	GM-CSF.	IL-10.	IL-1beta.	IL-6.	IL-8.	TNF-alpha.
S1	S1	A1,B1	7.26	0.39	0.89	0.66	2.69	0.7
S2	S2	C1,D1	0.7	0.46	0.37	0.31	0.39	1.16
S3	S3	E1,F1	0.26	0.42	2.03	2.56	1.24	1.47
S4	S4	G1,H1	0.83	1.71	2.94	1.24	1.75	2.12
S5	S5	A2,B2	2.48	2.47	0.35	2.04	1.03	2.44
S6	S6	C2,D2	5.81	0	1.1	2.51	0.71	2.05
S7	S7	E2,F2	6.73	4.71	2.18	2.4	2.28	4.04
S8	S8	G2,H2	N/A	10	2.67	4.04	5.14	N/A

S: Standard
